# XEN Gel Stent Failure Due to Luminal Obstruction

**DOI:** 10.18502/jovr.v19i3.9404

**Published:** 2024-09-16

**Authors:** Dilru C. Amarasekera, Vikram A. Shankar, Reza Razeghinejad

**Affiliations:** ^1^Wills Eye Hospital, Philadelphia, Pennsylvania, USA; ^2^Glaucoma Service, Wills Eye Hospital, Philadelphia, Pennsylvania, USA; ^4^Dilru Amarasekera: https://orcid.org/0000-0002-0210-3850

**Keywords:** Filtering Surgery, Glaucoma, Open Angle Glaucoma

## Abstract

**Purpose:**

To discuss four cases of post-operative XEN gel stent luminal obstruction in patients with primary open angle glaucoma.

**Case Report:**

Four eyes in three patients with primary open angle glaucoma who received XEN stent implantation were found to have luminal obstruction of their stents. Stent obstruction can mimic filtering bleb failures not responding to bleb needling and antimetabolite injections. These obstructions were suspected to result from fibrin clots, iris pigment granules, or breakdown products of intraocular inflammation or hemorrhage. Treatment options trialed in these patients included bleb needling, 5-fluorouracil injection, and YAG laser to the proximal end of the XEN. Ultimately, all four eyes required XEN explantation and alternative filtering surgery.

**Conclusion:**

XEN luminal obstruction is an important complication of stent placement that can ultimately lead to stent failure. Conservative measures such as laser or traditional bleb management may be considered before stent explantation or additional glaucoma surgery.

##  INTRODUCTION

Surgical management of primary open angle glaucoma (POAG) has evolved considerably over the last decade. While filtration procedures remain the mainstay of surgical management, numerous modifications to the traditional trabeculectomy have been developed. Minimally invasive glaucoma surgery (MIGS), though variably defined, is a safer, less invasive alternative to traditional glaucoma surgery. MIGS procedures may be combined with phacoemulsification cataract surgery and often spare the conjunctiva via an ab interno approach.^[1]^ Among these MIGS procedures is the XEN Gel Stent (Allergan INC, Dublin, Ireland), developed in 2015 and FDA approved for widespread use in 2016.^[2]^ The XEN Gel Stent is a hydrophilic tube composed of porcine gelatin that is placed in the anterior chamber and drains aqueous humor into the subconjunctival space.^[3,4]^

Several studies have compared the XEN gel stent with standard trabeculectomy and found similar overall rates of IOP reduction.^[5,6,7]^ One study showed that the XEN stent may have an improved side effect profile, with lower rates of anterior chamber shallowing and hyphema compared to trabeculectomy.^[6]^ Others have shown there to be no statistically significant difference in risk of failure or overall safety between XEN stent with mitomycin-C (MMC) and trabeculectomy with MMC.^[5,7]^

Given the relative novelty of the XEN stent, there exist few reports of post-operative complications. The most frequently described complications of stent placement are bleb encapsulation and fibrosis.^[8]^ Other reported complications include transient hypotony,^[8]^ choroidal detachment,^[9]^ implant exposure,^[10]^ late-onset bleb-associated endophthalmitis,^[11,12]^ and aqueous misdirection.^[13]^ A poorly understood and less frequently reported complication in the literature is XEN obstruction, where debris lodges in the lumen of the stent and ultimately results in stent failure.^[14,15,16]^

We present and analyze four unique cases of XEN luminal obstruction of varying etiologies.

**Table 1:** Summary of four cases of XEN luminal obstruction.


	**Time after surgery (months)**	**Material obstructing XEN**	**In-office intervention**	**Surgical intervention**
Case 1	18	Unidentified Yellow–white material	None	XEN explantation/CEIOL/tube shunt
Case 2	1	Unidentified Likely iris pigment	Bleb needling, 5-FU injection, Nd: Laser	XEN explantation/trab
Case 3	6	Iris stromal melanin	Bleb needling	XEN explantation/trab
Case 4	24	Unidentified Yellow material	None	XEN explantation/tube shunt
CEIOL, cataract extraction and intraocular lens placement; trab, trabeculectomy; 5-FU, 5-Fluorouracil				

**Figure 1:**
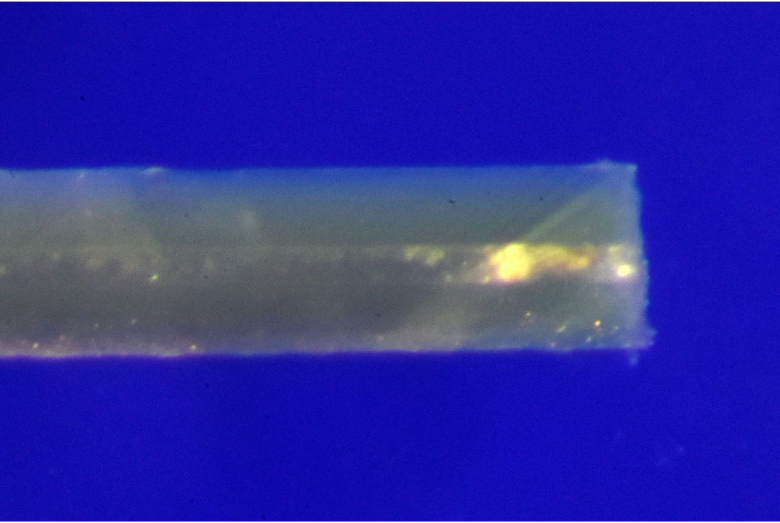
Explanted XEN stent from the right eye of patient one showing an irregular focus of yellow–white material blocking the lumen 100 micrometers from the ostium.

**Figure 2:**
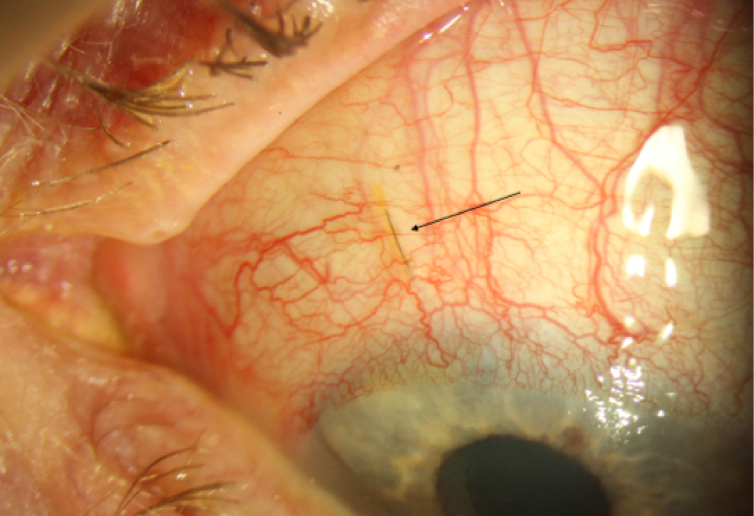
XEN stent (arrow) from the left eye of patient one showing a dark line within its lumen.

**Figure 3:**
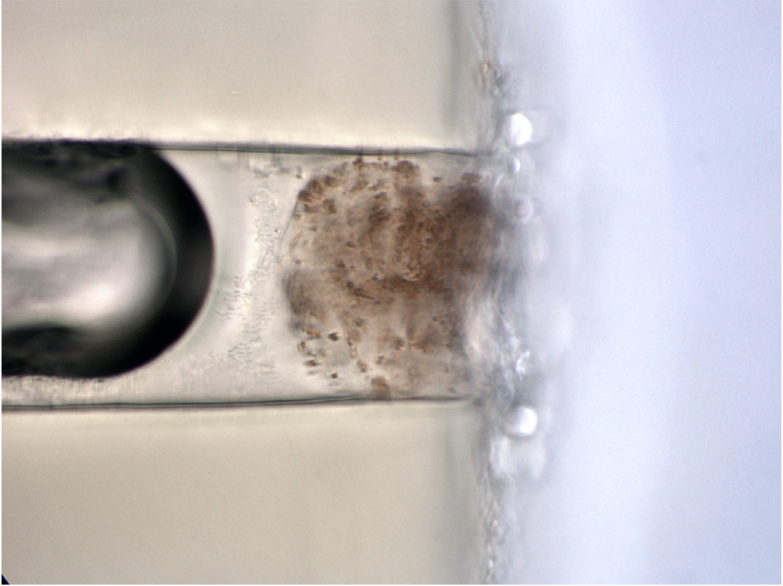
Luminal obstruction of a XEN stent with granules of pigment found to be iris stromal melanin.

**Figure 4:**
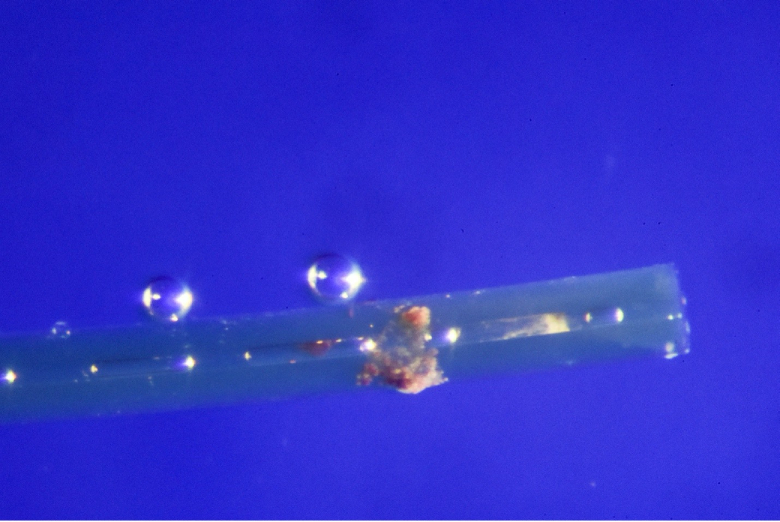
An explanted XEN stent showing luminal obstruction with yellow and red debris.

##  CASE REPORTS 

### Cases 1 and 2

A 62-year-old man with a history of POAG underwent ab-externo XEN gel stent placement with mitomycin-C (MMC) for uncontrolled intraocular pressure (IOP) in his right eye intermittently in the 30 to 40 mmHg range. Three weeks post-operatively, his IOP was well controlled at 10 mmHg. One week later, his IOP increased to 15 mmHg and his bleb showed evidence of early encapsulation. He received bleb-needling with 5-fluorouracil (5-FU) twice, each one week apart, with good response and a return of his IOP to 8 mmHg. His IOP remained stable in the low teens until one and a half years post-operatively when he returned to the office with an IOP of 32 mmHg. At that time his XEN stent was found to be in good position, but his bleb was shallow and scarred. Visual fields showed evidence of significant central field progression over the course of just four months. He was taken to the operating room for XEN explantation, cataract extraction with intraocular lens placement, and Baerveldt tube shunt placement. The explanted tube was sent to pathology, where it showed an irregular focus of yellow–white material blocking the lumen about 100 micrometers from the proximal ostium [Figure 1]. Despite thorough evaluation by ocular pathologists, the yellow material remained unidentifiable. Due to the small size of the XEN, sectioning was not feasible.

This same patient underwent ab-externo XEN gel stent placement in his contralateral eye for uncontrolled POAG with pressures in the mid-teens. In addition to stent placement, a surgical iridectomy was created intraoperatively to prevent XEN occlusion by iris as the XEN was minimally abutting the iris. In the early post-operative period, the IOP improved to 10 mmHg, however, one month post-operatively it was 30 mmHg. On examination he had a flat bleb, and a black line was visualized within the lumen of the XEN stent. There was no iris occlusion of the stent. Bleb needling and 5-FU injection failed to lower the IOP significantly. Nd: Laser was used to attempt to open the occluded XEN lumen. Five shots with 0.3–0.5mJ of power were applied to the proximal end of the XEN stent and seven shots were applied to the distal subconjunctival portion of the stent. Though his IOP improved to 14 mmHg following laser and medications including oral acetazolamide, he continued to have a flat bleb with a persistent dark line visualized in the lumen of the XEN. The patient was ultimately taken for XEN explantation and trabeculectomy of his left eye. The XEN broke during explantation, and this material was dislodged from the lumen and could not be analyzed microscopically [Figure 2].

### Case 3

A 68-year-old man with moderate POAG and an IOP of 22 mmHg on maximally tolerated medications underwent ab-externo XEN stent placement with MMC and good post-operative IOP control in the low teens. At six months post-operatively, his IOP increased to 20 mmHg and he was noted to have a scarring bleb. He was taken to the operating room for bleb needling versus trabeculectomy. The bleb needling resulted in no flow, and a trabeculectomy with XEN stent explantation was performed. Microscopic examination of the explanted XEN showed small granules of pigment consistent with iris stromal melanin causing blockage of the proximal end of the XEN [Figure 3].

### Case 4

The fourth case of XEN luminal obstruction occurred in a 70-year-old patient with severe bilateral normal tension glaucoma. He underwent ab externo XEN placement of his right eye. Post-operatively, the stent was found to lie against the iris without occlusion of the ostium. The IOP remained in the 7 to 10 mmHg range until two years post-operatively when the IOP rose to 14 mmHg with a flat bleb. He underwent XEN explantation and Baerveldt tube shunt placement. Microscopic evaluation revealed yellow material occluding the stent lumen, though its composition remained unknown [Figure 4].

##  DISCUSSION

Microinvasive glaucoma surgeries (MIGS) are minimally traumatic to ocular tissue and offer a superior safety profile and faster recovery than trabeculectomy and tube surgeries. Several of these devices target the trabecular meshwork or Schlemm's canal, while others offer IOP reduction by accessing the subconjunctival space. The XEN gel stent is a hydrophilic tube that has been widely acclaimed as a safe and effective alternative to traditional filtering surgery.^[5,7]^ Despite its notable safety profile, the XEN has been associated with several immediate and delayed post-operative complications.

As the XEN creates a conjunctival bleb, post-op bleb encapsulation and fibrosis are the most common complications of implantation, occurring in up to 32% of cases within one year.^[8]^ Bleb needling remains the procedure of choice for releasing scar tissue in the postoperative period and may be combined with MMC to produce durable reductions in IOP.^[17]^ In a large retrospective case series, Karimi and colleagues found that 40.9% of patients who had undergone XEN gel stent implantation required either physical or pharmacologic bleb management within 18 months.^[18]^

A rare complication of XEN gel stent placement involves blockage of the stent lumen with debris. Although this risk may be theoretically increased in patients with uveitic, pigmentary, or pseudoexfoliative glaucoma, the aforementioned four cases are of patients with POAG alone. The senior author of this study has previously described early postoperative occlusion with pigmented iris epithelium, and others have also demonstrated early occlusion with fibrin that may be treated with YAG fibrinolysis.^[19,20]^ In our series, one patient was found to have stent occlusion secondary to iris stromal melanin pigment and another patient had a dark line visualized in the stent lumen after surgical iridectomy. We hypothesize that the iridectomy led to dispersion of iris stromal pigment which ultimately blocked the lumen of this stent, although the material was dislodged prior to microscopic evaluation. Gillman and colleagues have previously described a patient with IOP elevation one month after stent placement, eventually finding cellular debris of unknown etiology obstructing its lumen.^[14]^ They speculated that post-operative hyphema and red blood cell breakdown were a threat to XEN gel stent patency. Clinically, these cases may be difficult to diagnose, as translucent cell debris may not be visible gonioscopically but can mimic a flat filtering bleb that does not respond to needling. These blockages may develop up to nine weeks after an intraocular hemorrhage and can cause delayed IOP elevation.^[14]^

XEN obstruction by the iris is an uncommon complication that may occur more frequently in patients with a shallow anterior chamber. In a large series from Karimi et al, 3.5% of patients experienced transient XEN occlusion by the iris.^[18,21]^ Improper placement of the stent including deeper approaches toward the iris root are also likely precipitating factors in iris occlusion. Both Argon laser iridoplasty and YAG iridotomy have been suggested to retract the surrounding iris or otherwise reopen the anterior segment ostium.^[16]^ Any sort of iris manipulation, however, may result in pigment release and XEN luminal obstruction.

It is unclear in some cases what exactly precipitates XEN obstruction. A case in the recent literature describing recurrent obstruction with fibrin in a patient with chronic uveitis suggests that prolonged inflammation increases a patient's risk.^[15]^ Both our patient who experienced bilateral stent failure and our final patient experienced stent obstruction by a yellow–white material that was difficult to identify despite pathologic evaluation. This yellow material could represent a proteinaceous material or minerals of the aqueous humor.

At present, the first approach to a failing XEN is bleb needling.^[17]^ We suggest gonioscopy at the first signs of IOP elevation to identify possible iris tissue, clot, or fibrin obstructing the stent lumen. If identified, the patient may benefit from YAG laser to the proximal end of the stent. If no material is identified, we recommend YAG iridotomy, and if no improvement still, then bleb needling followed by YAG laser to the proximal end of the stent for possible microscopic occlusion. If the patient continues to have persistently elevated IOP, trabeculectomy or tube shunt should be performed with subsequent removal of the XEN stent to prevent stent exposure and endophthalmitis.^[22]^

In summary, in this article, the authors present four cases of XEN gel stent obstruction of varying etiologies [Table 1]. Stent obstruction can mimic filtering bleb failures not responding to bleb needling and antimetabolite injections. While uncommon, these obstructions may result from fibrin clots, iris pigment granules, or from breakdown products of intraocular inflammation or hemorrhage. Conservative measures such as laser or traditional bleb management may be considered prior to stent explantation or additional glaucoma surgery.

##  Financial Support and Sponsorship

None.

##  Conflicts of Interest

None.
